# Soluble RAGE and skeletal muscle tissue RAGE expression profiles in lean and obese young adults across differential aerobic exercise intensities

**DOI:** 10.1152/japplphysiol.00748.2022

**Published:** 2023-09-07

**Authors:** Edwin R. Miranda, Jacob T. Mey, Brian K. Blackburn, Alec B. Chaves, Kelly N. Z. Fuller, Ryan K. Perkins, Andrew T. Ludlow, Jacob M. Haus

**Affiliations:** ^1^School of Kinesiology, University of Michigan, Ann Arbor, Michigan, United States; ^2^Applied Health Sciences, University of Illinois at Chicago, Chicago, Illinois, United States; ^3^Department of Nutrition and Integrative Physiology, University of Utah, Salt Lake City, Utah, United States; ^4^Integrated Physiology and Molecular Metabolism, Pennington Biomedical Research Center, Baton Rouge, Louisiana, United States; ^5^Applied Health Sciences and Kinesiology, Humboldt State University, Arcata, California, United States; ^6^Sarah W. Stedman Nutrition and Metabolism Center, Duke Molecular Physiology Institute, Duke University, Durham, North Carolina, United States; ^7^Division of Endocrinology, Department of Pediatrics, University of Colorado Anschutz Medical Campus, Aurora, Colorado, United States; ^8^Department of Kinesiology, California State University Chico, Chico, California, United States

**Keywords:** advanced glycation endproducts, exerkines, inflammation

## Abstract

Nearly 40% of Americans have obesity and are at increased risk for developing type 2 diabetes. Skeletal muscle is responsible for >80% of insulin-stimulated glucose uptake that is attenuated by the inflammatory milieu of obesity and augmented by aerobic exercise. The receptor for advanced glycation endproducts (RAGE) is an inflammatory receptor directly linking metabolic dysfunction with inflammation. Circulating soluble isoforms of RAGE (sRAGE) formed either by proteolytic cleavage (cRAGE) or alternative splicing (esRAGE) act as decoys for RAGE ligands, thereby counteracting RAGE-mediated inflammation. We aimed to determine if RAGE expression or alternative splicing of RAGE is altered by obesity in muscle, and whether acute aerobic exercise (AE) modifies RAGE and sRAGE. Young (20–34 yr) participants without [*n* = 17; body mass index (BMI): 22.6 ± 2.6 kg/m^2^] and with obesity (*n* = 7; BMI: 32.8 ± 2.9 kg/m^2^) performed acute aerobic exercise (AE) at 40%, 65%, or 80% of maximal aerobic capacity (V̇o_2max_; mL/kg/min) on separate visits. Blood was taken before and 30 min after each AE bout. Muscle biopsy samples were taken before, 30 min, and 3 h after the 80% V̇o_2max_ AE bout. Individuals with obesity had higher total RAGE and esRAGE mRNA and RAGE protein (*P* < 0.0001). In addition, RAGE and esRAGE transcripts correlated to transcripts of the NF-κB subunit P65 (*P* < 0.05). There was no effect of AE on total RAGE or esRAGE transcripts, or RAGE protein (*P* > 0.05), and AE tended to decrease circulating sRAGE in particular at lower intensities of exercise. RAGE expression is exacerbated in skeletal muscle with obesity, which may contribute to muscle inflammation via NF-κB. Future work should investigate the consequences of increased skeletal muscle RAGE on the development of obesity-related metabolic dysfunction and potential mitigating strategies.

**NEW & NOTEWORTHY** This study is the first to investigate the effects of aerobic exercise intensity on circulating sRAGE isoforms, muscle RAGE protein, and muscle RAGE splicing. sRAGE isoforms tended to diminish with exercise, although this effect was attenuated with increasing exercise intensity. Muscle RAGE protein and gene expression were unaffected by exercise. However, individuals with obesity displayed nearly twofold higher muscle RAGE protein and gene expression, which positively correlated with expression of the P65 subunit of NF-κB.

## Graphical abstract

**Figure:**
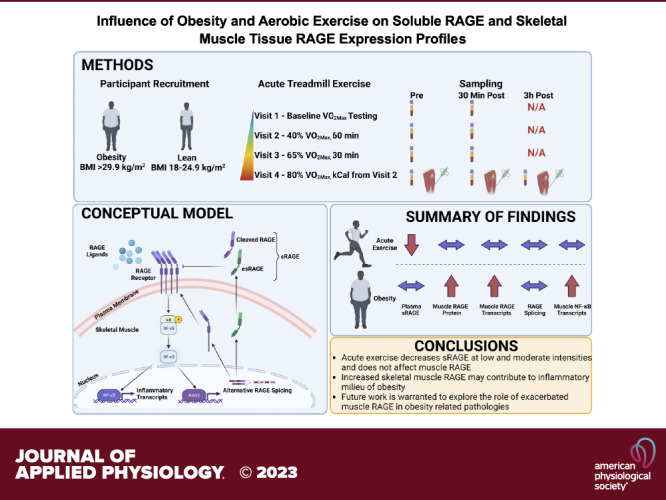

## INTRODUCTION

The receptor for advanced glycation endproducts (RAGE) is a transmembrane protein that promotes inflammation, oxidative stress, and metabolic dysfunction (1–3). Exacerbated RAGE expression as well as increased circulating concentrations of advanced glycation endproducts (AGEs), which act as the primary ligands for RAGE, have been well documented in a myriad of disease states, such as obesity (4–9), diabetes (10–14), cardiovascular disease (2, 15–23), aging (24–29), and nephropathy (13, 30–34).

Upon activation, RAGE promotes deterioration of cell and tissue function via inflammatory signaling and oxidative stress mechanisms such as NF-κB (2) and NADPH oxidase, respectively (35). Critically, NF-κB also promotes the transcription of RAGE itself, thus initiating a futile cycle of inflammation and oxidative stress. It is through the sustained propagation of this maladaptive mechanism that RAGE is implicated in promoting metabolic dysfunction including insulin resistance (36).

As a compensatory mechanism to the futile cycling of RAGE signaling, circulating RAGE proteins, termed soluble RAGE (sRAGE), are a heterogeneous pool of RAGE isoforms that lack the inter- and intracellular domains necessary to instigate downstream signal transduction and, thus, act as decoy receptors that block RAGE signaling. Endogenous secretory RAGE (esRAGE) (37) and cleaved RAGE (cRAGE) (38, 39) are the two isoforms of the total sRAGE pool. The esRAGE isoform is generated via alternative splicing in the RAGE premessenger RNA (pre-mRNA) at an alternative 3′ splice site in exon 9, which results in intron retention of intron 9 and skipping of exon 10 (37). The cRAGE isoform is generated via proteolytic cleavage of the RAGE receptor via matrix metalloproteases (MMPs).

Indeed, animal studies have demonstrated the efficacy of augmenting sRAGE concentrations for the prevention of obesity-related inflammation and metabolic dysfunction (7, 40, 41). In addition, the concentration of sRAGE isoforms has been repeatedly demonstrated to negatively correlate with the severity of obesity and glucose intolerance (10, 42–54). Several lines of evidence have also shown that sRAGE concentration can be increased by both acute and chronic interventions (55–63). For example, we have demonstrated that sRAGE concentrations are reduced following the consumption of a single high-fat meal in lean, healthy men (59), whereas sRAGE is reduced in mice fed a high-fat diet (5, 7). We and others have demonstrated that dietary or surgical interventions that result in weight loss increase sRAGE concentration and are related to better metabolic outcomes (62, 64, 65). In addition, several studies have demonstrated the efficacy of an aerobic exercise (AE) intervention to augment circulating sRAGE concentrations (55–57, 61, 66). However, the tissue source and mechanisms of sRAGE production following AE have not been evaluated and thus remain unknown. Skeletal muscle is responsible for >80% of insulin-stimulated glucose disposal and makes up a significant proportion of body mass (∼40% in active, lean healthy individuals) (67). Obesity promotes skeletal muscle inflammation and oxidative stress, which contribute to the pathogenesis of metabolic dysfunction including insulin resistance (68). AE is known to have an anti-inflammatory effect that is believed to be at least partly responsible for the resolution of metabolic dysfunction related to obesity (69). In addition, mechanisms related to skeletal muscle remodeling and energetic demand during AE may modulate the production of cRAGE and esRAGE in muscle, respectively. Specifically, the production of cRAGE is primarily generated by proteolytic activity of the matrix metalloproteinase ADAM10 (70). Interestingly, ADAM10 activity has been shown to be regulated by phosphorylation via calcium-sensitive kinases such as calcium calmodulin kinase (CAMK) and G protein-coupled receptors such as V2 that is activated by antidiuretic hormone (ADH), a hormone known to increase with exercise (38). In addition, calcium flux is necessary for muscle contraction and is a key signaling molecule for the induction of several molecular cascades necessary for exercise-induced muscle adaptation including CAMKII. Acute exercise is also a potent stimulus for modulation of alternative splicing in the muscle, which also plays a key role in the molecular transducers of exercise-mediated adaptations (71–74). The alternatively spliced esRAGE makes up the other predominant sRAGE isoform and is produced by skipping of exon 10, resulting in a protein lacking the transmembrane domain necessary for the protein to be embedded in the plasma membrane (75). However, the effect of AE on skeletal muscle RAGE protein expression and RAGE splicing in parallel with the measurement of the change in circulating sRAGE isoforms following acute AE has not been explored.

Therefore, the purpose of this study was twofold: *1*) to explore the potential ability of acute AE to enhance circulating RAGE, resolve skeletal muscle RAGE expression and related inflammatory signaling in lean individuals and individuals with obesity and *2*) to examine the effect of obesity on circulating sRAGE, skeletal muscle RAGE expression, alternative splicing, and related inflammatory signaling. We hypothesized that muscle RAGE protein and full-length transcripts would be higher, whereas esRAGE transcripts would be lower in muscle from individuals with obesity. We also hypothesized that circulating sRAGE isoforms would be lower in individuals with obesity but would be rescued by AE in an intensity-dependent manner.

## METHODS

### Study Design

**Figure 1.**
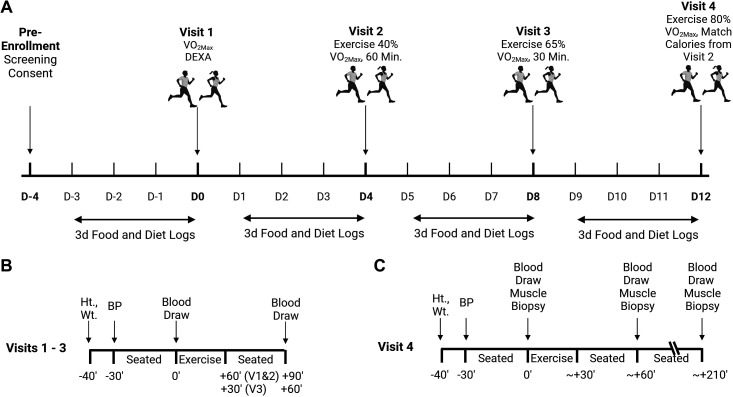
Study timeline. *A*: overall timeline of the study visits. Visits were separated by a washout period of at least 3 days. The last 3 days of the washout preceding each visit, diet, and food logs were provided, and participats were instructed to keep their diets and physical activity as consistent between visits as possible. *B*: sampling scheme for *visits 1*, *2*, and *3* describing the timing of collection of height (Ht.), weight (Wt.), blood pressure (BP), and blood samples. *C*: sampling scheme for *visit 4* describing the timing of collection of height (Ht.), weight (Wt.), blood pressure (BP), blood samples, and muscle biopsies. Figure was generated by BioRender. DEXA, dual X-ray absorptiometry; V̇o_2max_, maximal aerobic capacity.

The current investigation is a retrospective analysis of a larger study that consisted of four sequential visits and has been previously described, although this is the first reporting of circulating sRAGE isoforms and skeletal muscle RAGE outcomes (76, 77). Briefly, all participants provided verbal and written informed consent before participation. Baseline measures included height, weight, body composition via dual X-ray absorptiometry (DXA), and maximal aerobic capacity (V̇o_2max_). Participants returned to the laboratory on three subsequent occasions, each of which was separated by at least three days so as not to induce a training effect of the exercise. To control habitual diet and lifestyle habits, participants completed 3-day dietary records and physical activity logs. Participants were instructed to replicate their reported lifestyle and behavior for 3 days before each subsequent visit. Participants were also instructed to abstain from vigorous exercise, alcohol consumption 48 h before each visit, and caffeine consumption 24 h before each visit. Participants were asked to arrive at each visit by sedentary means (i.e., car, public transportation, etc.) and to have fasted for at least 12 h. Testing of all participants was done in the early morning (0600–0900 h) to account for any diurnal variations in outcome measures and to minimize the participants’ burden of fasting duration. During these visits participants performed treadmill exercise at 40% of their V̇o_2max_ for 60 min (*Visit 2*), 65% of their V̇o_2max_ for 30 min (*Visit 3*), and finally, 80% of their V̇o_2max_ until energy expenditure matched that of the AE bout performed at 40% V̇o_2max_ (*Visit 4*). The 65% V̇o_2max_ bout was not intentionally matched for calories (means ± SD kcals by visit: *V2*, 377.0 ± 96.5; *V3*, 315.7 ± 77.7; *V4*, 376.5 ± 21.6), but the ∼61 kcal lower expenditure during this bout is unlikely to be of consequence. During all exercise bouts, participants’ expired air was collected and analyzed via indirect calorimetry (PARVOMedics, Salt Lake City, UT) to ensure that the appropriate workload was being attained throughout the protocol. All experimental protocols were approved by the Institutional Review Board at the University of Illinois of Chicago (IRB Approval No.: 2015-0127). A timeline for the clinical procedures is presented in Fig. 1.

### Participants

Fifteen lean, healthy (LH) adults and seven adults with obesity (OB) volunteered to participate in the study. Participants were screened for eligibility using the following inclusion criteria: 18–35 yr of age, body mass index (BMI) between 18 and 26 kg/m^2^ (LH) or >29.9 kg/m^2^ (OB). Participants were excluded if they were current smokers, quit smoking within the past year, and if they were previously diagnosed with any major disease such as diabetes, cardiovascular disease, kidney disease, major depression, high blood pressure, or high blood cholesterol. Participant characteristics are presented in Table 1. However, despite the OB group being mildly obese (class I obesity), they did present with elevated fasting insulin that drove a significantly elevated homeostatic model assessment for insulin resistance (HOMA-IR) in the OB compared with LH participants suggesting the presence of metabolic dysfunction.

**Table 1.** Participant characteristics

Variable, Units	LH, *n* = 17	OB, *n* = 7	*P*
Age, yr	25 ± 3.9	28 4.5	0.16
Sex, %F	41%	43%	
Weight, kg	68.4 ± 9.9	96.2 ± 17.3	**<0.0001**
BMI, kg/m^2^	22.6 ± 2.6	32.8 ± 2.9	**<0.0001**
Lean mass, %	75.6 ± 5.6	58.9 ± 5.4	**<0.0001**
Fat mass, %	23.8 ± 1.4	38.3 ± 2.8	**<0.0001**
Glucose, mg/dL	91.5 ± 2.1	89.1 ± 4.2	0.585
Insulin, mU/L	4.9 ± 0.5	11.6 ± 3.4	**0.005**
HOMA-IR	1.1 ± 0.1	2.5 ± 0.7	**0.006**
Systolic blood pressure, mmHg	115 ± 7.9*	117 ± 10.4	0.999
Diastolic blood pressure, mmHg	68.6 ± 7.3	67.4 ± 7.8	0.745
V̇o_2max_, mL/kg/min	48.0 ± 7.0*	35.5 ± 6.8	**0.001**
V̇o_2max_, L/min	3.3 ± 0.88	3.4 ± 0.9	0.792

Lean healthy (LH) individuals and individuals with obesity (OB) were matched for age. By design, weight and body mass index (BMI) were significantly greater in the OB compared with LH group. *Data are presented as means ± SD and were compared via unpaired *t* test or Mann–Whitney *U* test if data are non-normally distributed. Bold values are statistically significant (*P* < 0.05). Sample sizes for glucose, insulin, and HOMA-IR are *n* = 15 for LH and *n* = 5 for OB. Significance was set to *P* < 0.05. AU, arbitrary units; HOMA-IR, homeostatic model assessment for insulin resistance; V̇o_2max_, maximal aerobic capacity.

### V̇o_2max_ and AE Tests

V̇o_2max_ was determined using a treadmill ramp protocol during which the participants ran at a self-selected speed while the treadmill grade increased 2% every 2 min, until volitional fatigue was reached. Participants were consulted on the selection of the speed with the goal of reaching their max at 8–12 min. Expired air was collected for the duration of the test and was analyzed via the PARVO Medics metabolic cart (Salt Lake City, UT). Heart rate was also monitored via Polar heart rate monitors fitted to the participants’ chest before testing. Rating of perceived exertion (RPE) on the Borg scale (6–20) was also assessed at the end of each 2-min interval. V̇o_2max_ was achieved if the subjects met three of the following four criteria: a lack of obvious increase in oxygen consumption (V̇o_2_) despite an increase in workload, an RPE > 17, respiratory exchange ratio > 1.1, and a heart rate > 85% age-predicted maximal heart rate. Before each subsequent visit, American College of Sports Medicine metabolic equations were used to derive the estimated treadmill settings (speed and grade) as a starting intensity for each bout. Participants’ expired air was collected throughout the acute exercise test to monitor and confirm that the appropriate V̇o_2_ was obtained during the exercise. Adjustments to speed and incline were made as necessary throughout to achieve and maintain the goal V̇o_2_ by a trained exercise physiologist with experience in conducting V̇o_2max_ testing.

### Blood Collection and Processing

Blood was collected via venipuncture into ethylenediaminetetraacetic acid (EDTA)-coated vacutainers before (Pre) and 30 min after (Post) each exercise bout. For the last visit (AE at 80% V̇o_2max_), a blood draw was also taken 3 h after exercise (3 h) which coincided with the last muscle biopsy sampling (described under *Skeletal Muscle Biopsy and Tissue Homogenization*). To account for blood volume shifts (78) that occur with rapid changes in body position (e.g., transition from standing or exercising to sitting or lying supine), participants were asked to sit quietly for 30 min before each blood draw. Blood collected in EDTA tubes was immediately centrifuged at 3,000 rpm for 10 min at 4°C (C) to isolate plasma that was then aliquoted and stored at −80°C until further analysis. Pre-exercise plasma samples were later analyzed for glucose via YSI glucose analyzer 2300 (YSI Life Sciences, Yellow Springs, OH), and insulin was measured using ELISA (90095, Crystal Chem, Elk Grove Village, IL).

### Determination of sRAGE Isoforms

Total sRAGE concentrations were measured in plasma samples by commercial ELISA (DRG00, R&D Systems, Minneapolis, MN) as per the manufacturer’s protocol. This measure of total sRAGE is a heterogeneous combination of cleaved (cRAGE) and alternatively spliced (esRAGE) sRAGE isoforms. The kit for total sRAGE uses a monoclonal antibody raised against the extracellular domain of RAGE, comprising amino acids 24–344, and therefore captures all sRAGE isoforms. Plasma esRAGE concentrations were measured by commercial ELISA (As One International, Mountain View, CA), which utilizes a monoclonal antibody raised against human esRAGE that includes an epitope consisting of amino acids 332–347. Plasma cRAGE concentrations were subsequently calculated by subtracting the esRAGE value from the total sRAGE as previously described (42). All samples were analyzed in duplicate.

### Skeletal Muscle Biopsy and Tissue Homogenization

Participant burden during this study was significant and particularly affected our ability to recruit and complete individuals for the OB group. We anticipated that this may be the case; therefore, in attempt to limit participant burden and because we hypothesized that the highest intensity exercise would have the largest effect on muscle RAGE and circulating RAGE, we only performed muscle biopsies on the final visit. Briefly, skeletal muscle biopsies were taken from the m. vastus lateralis at pre, 30-min post- and 3-h postexercise under local anesthetic (Lidocaine, 2% without epinephrine) as previously described (76). These time points were selected because we hypothesized that RAGE shedding in the case of cRAGE production and alterations in splicing and exocytic release in the case of esRAGE production by the muscle would happen rapidly following exercise (within hours). The extracted muscle tissue was quickly cleaned of all visible connective tissue, fat, and blood. Portions of the samples designated for protein extraction for Western blotting experiments were then flash frozen in liquid nitrogen. Portions of the samples designated for RNA isolation were placed in RNALater (Invitrogen) before freezing in liquid nitrogen. Samples were then stored at −80°C until analysis. Approximately 10 mg of muscle tissue was weighed in a custom-built freezer and homogenized with ceramic beads (Lysing Matrix D; FastPrep-24; MP Bio, Santa Ana, CA) in 20 volumes of ice-cold 1X Cell Lysis Buffer (No. 9803, Cell Signaling Technology, Danvers, MA) supplemented with 1X Protease/Phosphatase Inhibitor Cocktail (No. 5872, Cell Signaling Technology, Danvers, MA). Protein concentration for each sample homogenate was determined by a commercially available bicinchoninic acid (BCA) protein assay kit (Pierce, Rockford, IL).

### Western Blotting

Aliquots of muscle homogenate or cell lysates containing 10–20 µg of total protein were diluted in equal volumes of 2X Laemmli Buffer (BioRad, 1610737, Hercules, CA) with 5% β-mercaptoethanol (βME) (BioRad, 1610710) before heating at 90°C for 10 min. Denatured samples were brought to room temperature, loaded onto 10% precast Criterion TGX gels (5671033, BioRad), and separated by SDS–PAGE at 200 V for 50 min. Separated proteins were then transferred to nitrocellulose membranes via Transblot Turbo semidry transfer system for 11 min (BioRad). Membranes were then blocked with protein-free blocking buffer (PFBB, 92780003, Li-Cor, Lincoln, NE) for 1 h at room temperature with gentle rocking. After blocking, membranes were incubated overnight at 4°C with a primary antibody raised against RAGE (1:500, ab3611, Abcam) or Toll-like receptor 4 (TLR4) (1:1,000, sc93072, Santa Cruz Biotechnology). RAGE antibody was validated in-house via siRNA-mediated knockdown (Supplemental Fig. S3), and TLR4 antibody was previously validated using similar methods (79). The next day, membranes were removed from primary antibody solutions, washed, and incubated with a fluorophore-conjugated secondary antibody (1:20,000, Li-Cor, Lincoln, NE) diluted in PFBB + 0.1% Tween-20 for 1 h at room temperature with gentle rocking while protected from light. The secondary antibody solution was subsequently removed, and the membranes were washed before scanning on an Odyssey CLx Imaging System (Li-Cor). After imaging was completed for the proteins of interest, membranes were incubated for 30 s in 5 mL of ponceau total protein stain. Ponceaus images were captured on a Bio-Rad ChemiDoc imaging cabinet (BioRad). Fluorescent and ponceau signals were both quantified on Image Studio software (V4.0.21; Li-Cor) using the Western blot quantification function. All target protein signals were made relative to the total protein stain acquired from the Ponceau staining.

### RNA Extraction and Reverse Transcription

RNA extraction was performed using Qiagen’s RNeasy kit following the kit protocol with modifications to increase yield for fibrous tissues such as muscle. Briefly, RNALater-fixed samples were homogenized in RNA lysis buffer with β-mercaptoethanol via bead homogenization as described under *Skeletal Muscle Biopsy and Tissue Homogenization* above. Samples were treated with 10 μL Proteinase K (Qiagen), incubated at 55°C for 10 min, and then centrifuged at 10,000 *g* for 3 min at room temperature. Supernatants were transferred to a sterile microfuge tube and 450 μL of ethanol was added to each sample and transferred on to the RNeasy spin columns. Ethanol extracts were collected via centrifugation at 9,000 *g* for 30 s at room temperature, and the protocol provided by Qiagen was then followed thereafter. Two microliters of each extraction was analyzed for RNA concentration via Nanodrop (Thermo Fisher). Average RNA concentration was 128 ± 9.9 ng/μL (means ± SE) for LH and 118 ± 17.7 ng/μL for OB samples. Reverse transcription was performed with iScript Advanced reverse transcriptase kit (BioRad) via manufacturer’s protocols to generate 150 ng of cDNA that was then diluted 1:4 with nuclease-free water.

### Droplet Digital PCR

Droplet digital PCR (BioRad) was used to quantify transcripts of RAGE, esRAGE, P65, and hypoxia inducible factor-1α (HIF-1α) as previously described (80). Primers were designed for each target using Roche’s universal probe library (UPL) assay design center (lifescience.roche.com). In brief, droplet digital polymerase chain reaction (ddPCR) assays were performed by combining 2 μL of cDNA (3.75 ng) with ddPCR mix for probes, no dUTP (BioRad), along with appropriate probes, primers, and nuclease-free water, yielding a 20 μL reaction. To control for a small amount of background signal in the ddPCR, a triplicate of no-template negative controls in which the cDNA was substituted for nuclease-free water was performed alongside each of the assays and subtracted from the final signal. The 20 μL reaction was then combined with 70 μL of droplet generation oil for probes (BioRad), and droplets were generated using a droplet generator (BioRad). A total of 40 µL of the resultant droplet suspension was then carefully pipetted onto a 96-well plate, which was then sealed and placed in a thermocycler where 40 cycles of PCR were performed. The droplets were then analyzed using a QX200 Droplet Digital PCR system (BioRad) by counting the droplets positive for fluorescein amidite fluorescent probes. Transcript copy number was corrected for nonspecific signal by subtracting the number of copies detected in the no-template control samples. All transcript data are presented as copy number per nanogram cDNA input. A custom primer pair was designed to capture all protein-coding RAGE transcripts (Total RAGE), and another primer pair was designed to only capture esRAGE isoforms (IDT). This was achieved by targeting the forward primer for the “Total RAGE” transcript to span the junction of exons 3 and 4, whereas the reverse primer was targeted to exon 5. This segment is present in all protein-coding RAGE transcripts. To detect the esRAGE transcript, the forward primer was designed to detect the unique intron 9 retention event, and the reverse primer was targeted to exon 11. UPL probes were used for each of the targets, and the sequences are provided for those and the corresponding primers in Table 2. The percent of intron 9 being spliced out and thus directing RAGE splicing to form full-length RAGE transcripts was calculated by the following equation:

$$\%\;Spliced\;Out=\;\frac{Total\;RAGE\;-esRAGE\;}{Total\;RAGE}\;\times \;100$$

**Table 2.** Sequences of primers and probes used for ddPCR

Target	Ref Number	Forward Primer (5′–3′)	Reverse Primer (5′–3′)	UPL Probe Number	UPL Probe Sequence
HIF-1α	NM_001530.4	GATAGCAAGACTTTCCTCAGTCG	TGGCTCATATCCCATCAATTC	64	CCAGGCTG
NF-κB p65	NM_021975.4	ACCGCTGCATCCACAGTT	GATGCGCTGACTGATAGCC	47	ACACTGGA
Total RAGE	NM_001136.5	TCCGTGTCTACCAGATTCCTG	GACACACATGTCCCCACCTT	34	AGAAGGCAG
esRAGE	NM_001206840	GGACCAGGGAACCTACAGC	TGACTTTATCAAACCCCTCACC	57	GGCCCCAG

Primers were synthesized by IDT and were designed by aligning sequences in Snap Gene. Universal probe library (UPL) probes used were all conjugated to fluorescein amidite fluorophores with the exception of endogenous secretory receptor for advanced glycation endproducts (esRAGE), which was conjugated to a hexachlorofluorescein fluorophore.

On rare occasions, esRAGE copies were higher than total RAGE transcripts, yielding negative values for % spliced out and were excluded. To gain a more inclusive view of the data, we also calculated the ratio of esRAGE:Total RAGE transcripts, which resulted in similar findings as % spliced out (Supplemental Fig. S1).

### Statistics

All figures were made, and statistical analysis was run in GraphPad Prism (Version No. 9.4.0). Individual data points are provided for transparency, and bars represent the mean values and error bars represent the standard deviation (SD). Mixed-effects analysis was used to analyze Western blot, PCR, and sRAGE data with post hoc analyses utilizing Bonferroni correction. Data were checked for normality using Shapiro–Wilk’s test. Pearson’s or Spearman’s correlations were used for correlative analysis depending on the normality of the outcomes assessed. Linear regressions were used to determine the influence of clinical variables on outcome measures. The *P* value for statistical significance was set at *P* < 0.05.

## RESULTS

### Participant Characteristics

Participant baseline characteristics are presented in Table 1. Table 1 data describes all participants who at least completed the exercise, and blood draws in the second visit (40% V̇o_2max_, *n* = 7). Unfortunately, two individuals in the OB group had dropped out before the final visit. In addition, some missing data exists for blood parameters taken throughout the study due to the difficulty of venous access for blood draws in some participants in the OB group. By design, the OB group demonstrated a significantly higher BMI and was found to be less fit than the LH group based on their relative V̇o_2max_. While mildly the OB group was considered class I obese, they nevertheless possessed significantly elevated fasting insulin and HOMA-IR values compared with the LH group (Table 1).

### Soluble RAGE Isoforms Are Differentially Regulated by Acute AE Intensity but Not Altered with Obesity

**Figure 2.**
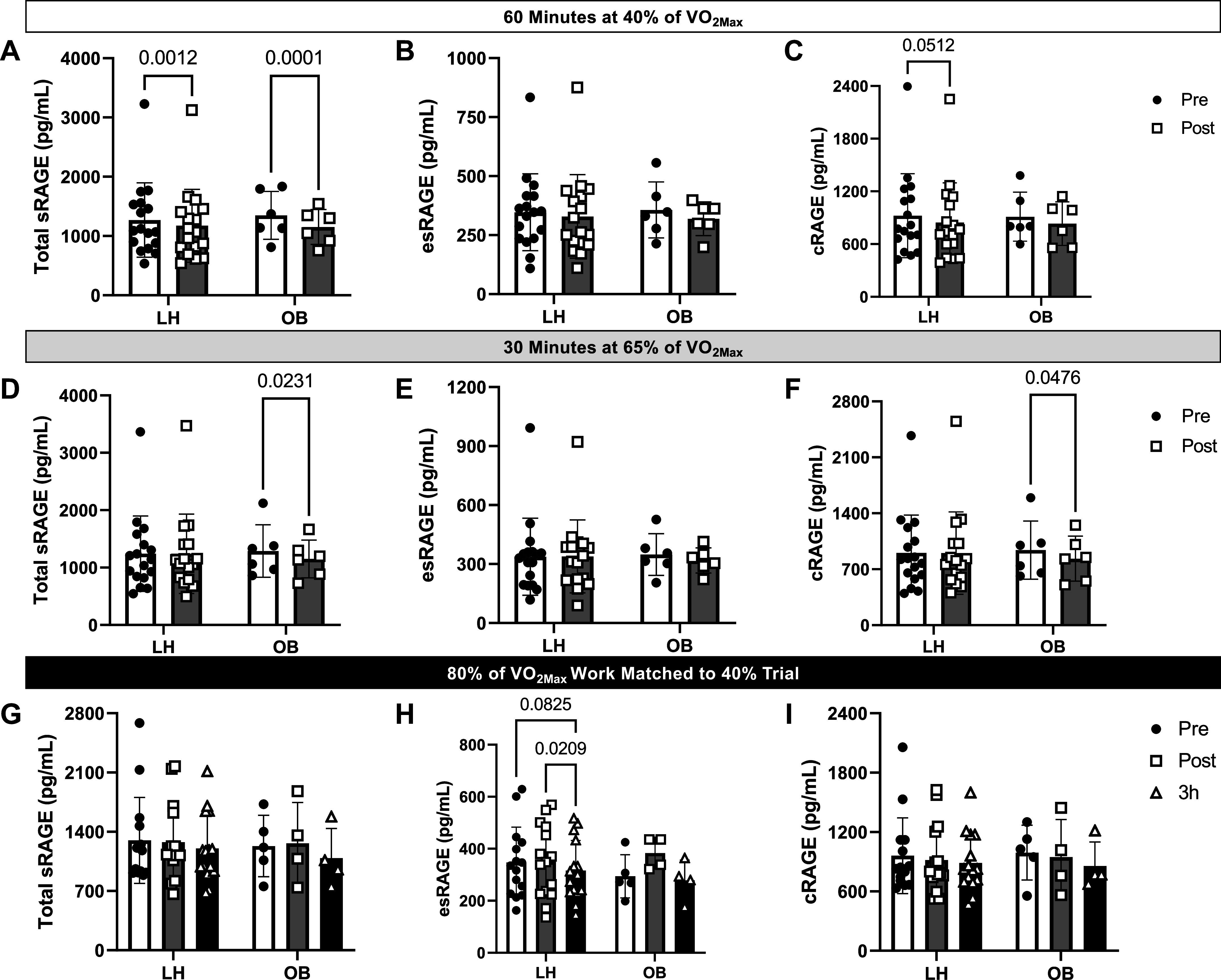
Effect of obesity and acute aerobic exercise on soluble receptor for advanced glycation endproducts (sRAGE) isoforms. *A*–*C*: plasma concentrations of total sRAGE, endogenous secretory RAGE (esRAGE), and cleaved RAGE (cRAGE) before (Pre) and 30 min after (Post) treadmill exercise performed at 40% of participants’ V̇o_2max_ for 60 min. *D*–*F*: plasma concentrations of total sRAGE, esRAGE, and cRAGE before (Pre) and 30 min after (Post) treadmill exercise performed at 65% of participants’ V̇o_2max_ for 30 min. *G*–*I*: plasma concentrations of total sRAGE, esRAGE, and cRAGE before (Pre), 30 min (Post), and 3 h (3 h) after treadmill exercise performed at 80% of participants’ V̇o_2max_ until calorie expenditure matched that which they obtained during the 40% exercise bout. Data are represented as means ± SD, and individual data points are plotted about the mean. Data were analyzed via mixed-effects analysis with Bonferroni correction used for post hoc analyses. *P* values for pairwise comparisons are shown when main effects reached significance (*P* < 0.05). LH, lean healthy adults; OB, adults with obesity; V̇o_2max_, maximal aerobic capacity.

In Fig. 2*A*, baseline sRAGE isoforms were similar between the LH and OB groups. AE decreased total sRAGE in both the LH and OB groups (Fig. 2*A*), whereby the decrease was greater in the OB compared with the LH group (*P* for interaction = 0.040; Supplemental Fig. S2*A*). There was no effect of exercise at 40% of V̇o_2max_ on esRAGE, whereas cRAGE was trending toward a decrease in the LH group only (*P* = 0.051, Fig. 2*C*). AE at 65% of V̇o_2max_ decreased total sRAGE only in the OB group (*P* = 0.023, Fig. 2*D*), which appeared to be attributed to decreased cRAGE (*P* = 0.048, Fig. 2*F*). AE at 80% V̇o_2max_ only resulted in trends for decreased esRAGE 3-h post-AE in the LH group (*P* = 0.083, Fig. 2*H*). We did not observe any effect of sex on any sRAGE isoforms at baseline (Supplemental Fig. S4, *D*–*F*).

**Figure 3.**
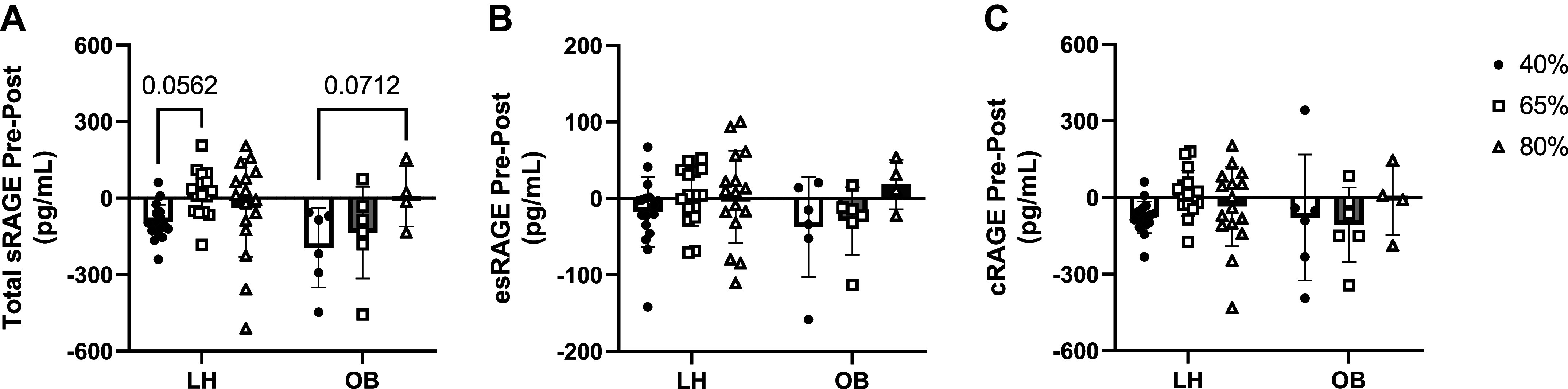
Effect of acute exercise intensity on soluble receptor for advanced glycation endproducts (sRAGE) isoforms in lean healthy adults (LH) and adults with obesity (OB). Change in plasma concentrations of total sRAGE (*A*), endogenous secretory RAGE (esRAGE, *B*), and cleaved RAGE (cRAGE, *C*) were calculated by subtracting values obtained at baseline (Pre) from values obtained 30 min after (Post) treadmill exercise performed at 40% of participants’ V̇o_2max_ for 60 min, 65% of V̇o_2max_ for 30 min, or 80% of participants’ V̇o_2max_ until calorie expenditure matched that which they obtained during the 40% exercise bout. Data are represented as means ± SD, and individual data points are plotted about the mean. Data were analyzed via 2-way ANOVA with Bonferroni correction used for post hoc analyses. *P* values for pairwise comparisons are shown when main effects approached significance (*P* < 0.05). V̇o_2max_, maximal aerobic capacity.

We next calculated the change of sRAGE isoforms (Pre − Post) across all exercise intensities to determine whether there was an intensity-dependent effect of exercise (Fig. 3). The change in total sRAGE tended to increase with increased intensity (main effect of time: *P* = 0.03; interaction effect: *P* = 0.13; Fig. 3*A*). This general pattern was also recapitulated for the changes in esRAGE (Fig. 3*B*) and cRAGE (Fig. 3*C*), although these effects were not statistically significant or trending toward significance. Importantly, the interpretation of these data did not change after the exclusion of data that were identified as outliers (Supplemental Fig. S5). Therefore, we have presented the full available dataset in the main manuscript.

### RAGE Expression Is Not Affected by AE but Is Exacerbated in Skeletal Muscle with Obesity

**Figure 4.**
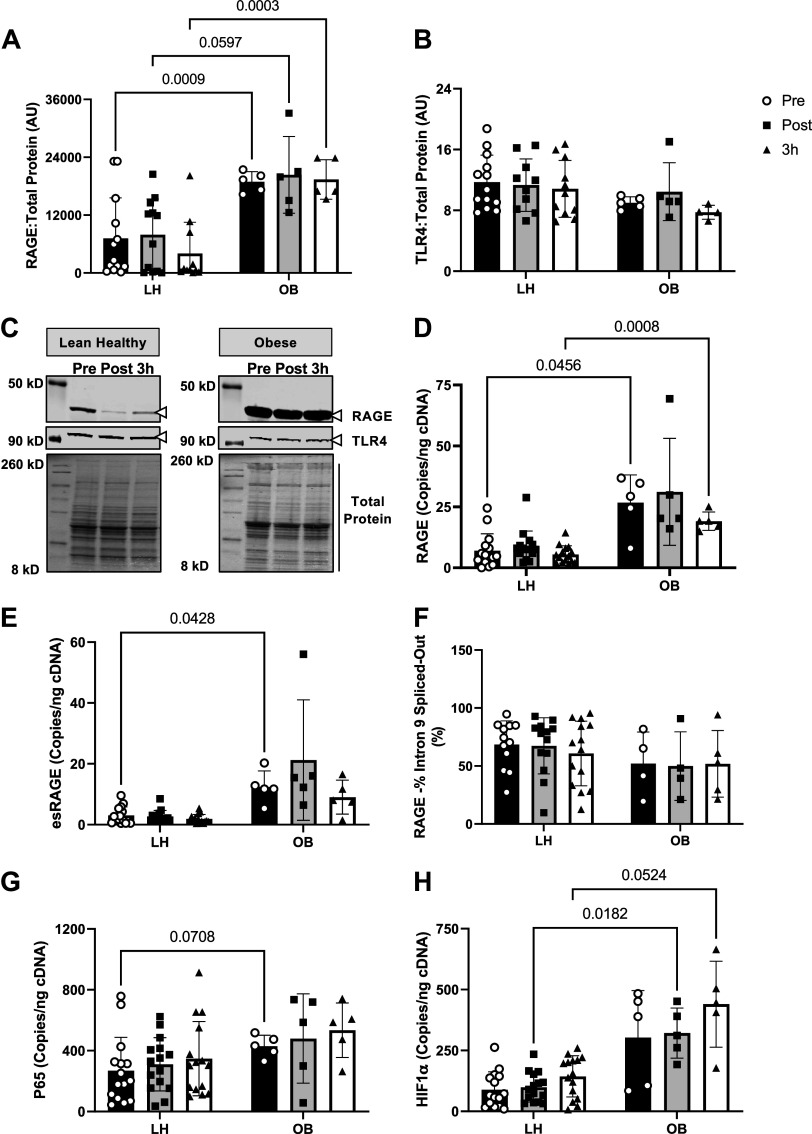
Effect of obesity and acute exercise on receptor for advanced glycation endproducts (RAGE) expression in muscle biopsy samples. Muscle biopsies taken from the vastus lateralis before (pre), 30 min after (post), or 3 h after (3 h) exercise at 80% of V̇o_2max_ until calorie expenditure matched that which they obtained during the 40% exercise bout and then subsequently probed for RAGE protein (*A*), TLR4 protein (*B*), total RAGE transcripts (*D*), endogenous secretory RAGE (esRAGE) transcripts (*E*), percent of intron 9 spliced out of RAGE transcripts (*F*), P65 transcripts (*G*), and HIF-1α transcripts (*H*). *C*: representative Western blot images probing for RAGE, TLR4, and total protein in muscle samples. Data are presented as means ± SD, and individual data points are plotted about the mean. Data were analyzed via mixed-effects analysis with Bonferroni correction used for post hoc analyses. *P* values for pairwise comparisons are shown when main effects reached significance (*P* < 0.05). AU, arbitrary units; LH, lean healthy adults; OB, adults with obesity; V̇o_2max_, maximal aerobic capacity.

In contrast to our hypothesis, we did not find any effect of acute AE at 80% of V̇o_2max_ on RAGE protein expression, full-length RAGE transcripts or RAGE splicing with AE (Fig. 4). These findings are in line with our sRAGE data which did not demonstrate a robust increase in sRAGE concentrations at any of the AE intensities.

However, RAGE protein abundance in muscle from individuals with obesity was nearly twofold higher compared with LH group (Fig. 4*A*). Transcripts of the active subunit of NF-κB, P65, and HIF-1α, which is downstream of P65 transcriptional regulation were also elevated in OB muscle (Fig. 4, *G*–*H*). We did not observe any effect of sex on RAGE protein, RAGE transcripts, or esRAGE transcripts (Supplemental Fig. S4, *A*–*C*). Interestingly, baseline levels of RAGE protein, RAGE transcripts, and P65 transcripts did not correlate with changes in sRAGE isoforms (Supplemental Table S2). As a part of RAGE signal transduction, RAGE forms homodimers with itself and heterodimers with other inflammatory receptors when activated by different ligands such as AGEs. TLR4 is another inflammatory pattern recognition receptor, which is known to form heterodimers with RAGE (81). However, unlike RAGE, TLR4 protein abundance was not affected by obesity or AE (Fig. 4*B*).

**Figure 5.**
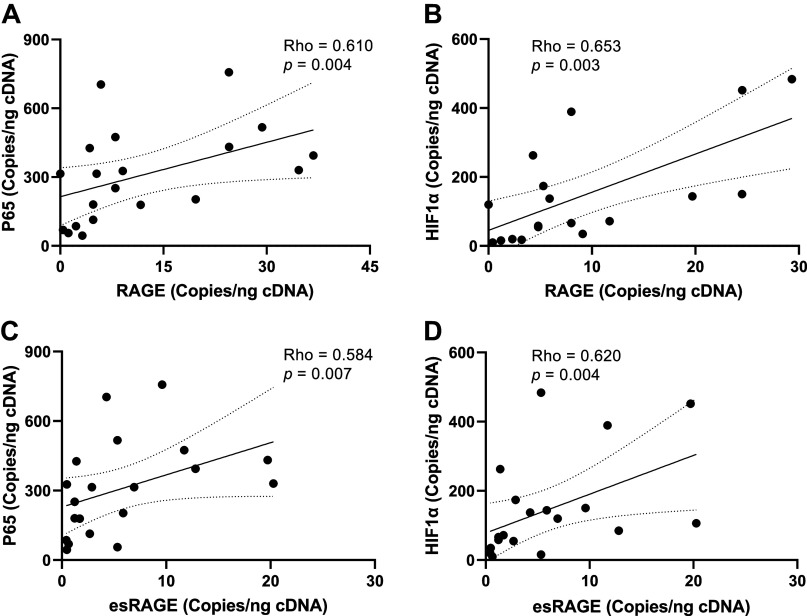
Correlations between total receptor for advanced glycation endproducts (RAGE) and endogenous secretory RAGE (esRAGE) and inflammatory transcripts. Spearman correlations were performed to assess the correlation between total RAGE transcripts and transcripts of the P65 subunit of NF-κB at baseline (*A*), correlation between total RAGE and HIF-1α transcripts at baseline (*B*), correlation between esRAGE transcripts and transcripts of the P65 subunit of NF-κB at baseline (*C*), and the correlation between esRAGE and HIF-1α transcripts at baseline (*D*). Linear regression line with dotted lines representing the 95% confidence intervals are presented.

To explore if exacerbated RAGE protein abundance in muscle was related to alternative splicing favoring the production of full-length RAGE transcripts over esRAGE transcripts, we designed a set of primers that are complementary to measuring full-length RAGE and another set that are specifically complementary to esRAGE transcripts (Table 2). Using ddPCR, we quantified the number of each of these transcripts per nanogram cDNA input. However, contrary to our hypothesis both full-length and esRAGE transcripts were elevated by approximately twofold in OB muscle (Fig. 4). In addition, the calculated percentage of transcripts from which exon 10 had been spliced out to generate esRAGE (% spliced out) was also not different between groups. Linear regression analysis were then deployed to determine if any parameters beyond BMI and body fat percentage explained variance in RAGE protein (Table 3) or RAGE transcripts (Table 4). BMI and body fat percentage did not significantly explain the variance in muscle RAGE protein. Conversely, fasting glucose, insulin, and HOMA-IR did significantly explain RAGE protein variance (Table 3). However, contrary to what we expected, both fasting glucose (β = −2,050, *P* = 0.0095) and insulin (β = −16,325, *P* = 0.0438) were negatively related to RAGE protein abundance. Interestingly, HOMA-IR was positively related to RAGE protein abundance (β = 77,076, *P* = 0.0456). Lastly, both full-length and esRAGE transcripts were positively correlated to p65 and HIF-1α transcripts (Fig. 5). Together, these data suggest that the increased RAGE protein abundance in muscle from individuals with obesity is being driven primarily by a global upregulation of RAGE gene expression.

**Table 3.** Multiple regression analysis for baseline RAGE protein abundance in muscle

Dependent Variable: RAGE Protein					
Variable	Estimate	Standard Error	95% CI (Asymptotic)	|t|	*P* Value
Intercept	194,718	57,747	58,167 to 331,269	3.372	**0.0119**
SBP, mmHg	15,608	19,375	−30,207 to 61,423	0.806	0.4470
DBP, mmHg	29,184	38,709	−62,349 to 120,717	0.754	0.4755
MAP	−44,136	57,962	−181,194 to 92,922	0.762	0.4712
V̇o_2max_, mL/kg/min	−44	412	−1,017 to 929	0.107	0.9180
BMI, kg/m^2^	−1,093	773	−2,919 to 734	1.415	0.2001
Fat mass, %	356	571	−993.6 to 1,705	0.623	0.5528
Lean mass, %	−994	350	−1,821 to −167	2.843	**0.0249**
Blood glucose, mg/dL	−2,050	579	−3,419 to −680	3.539	**0.0095**
Insulin, mU/L	−16,325	6,650	−32,049 to −601	2.455	**0.0438**
HOMA-IR	77,076	31,752	1,995 to 152,158	2.427	**0.0456**
*R* ^2^	0.85				

A multiple linear regression analysis was performed to determine predictive independent variables for skeletal muscle receptor for advanced glycation endproducts (RAGE) protein abundance. All data were found to be normally distributed. *P* for significance <0.05. BMI, body mass index; 95% CI, 95% confidence interval; DBP, diastolic blood pressure; HOMA-IR, homeostatic model assessment for insulin resistance; MAP, mean arterial pressure; SBP, systolic blood pressure; |t|, t statistic.

**Table 4.** Multiple regression analysis for baseline RAGE gene expression in muscle

Dependent Variable: RAGE Transcripts					
Variable	Estimate	Standard Error	95% CI (Asymptotic)	|t|	*P* Value
Intercept	188.60	90.90	−21.02 to 398.20	2.075	0.0717
SBP, mmHg	21.77	31.51	−50.89 to 94.44	0.691	0.5091
DBP, mmHg	41.58	62.83	−103.30 to 186.50	0.662	0.5267
MAP	−63.80	94.10	−280.80 to 153.20	0.678	0.5169
V̇o_2max_, mL/kg/min	0.33	0.66	−1.20 to 1.90	0.493	0.6351
BMI, kg/m^2^	0.16	1.28	−2.78 to 3.10	0.127	0.9022
Fat mass, %	−0.0042	0.95	−2.20 to 2.19	0.004	0.9966
Lean mass, %	−0.83	0.51	−2.02 to 0.35	1.616	0.1447
Blood glucose, mg/dL	−1.62	0.97	−3.86 to 0.61	1.669	0.1337
Insulin, mU/L	−17.84	11.19	−43.66 to 7.98	1.594	0.1497
HOMA-IR	90.72	53.47	−32.59 to 214.0	1.697	0.1282
*R* ^2^	0.75				

A multiple linear regression analysis was performed to determine predictive independent variables for skeletal muscle receptor for advanced glycation endproducts (RAGE) gene expression. All data were found to be normally distributed. *P* for significance <0.05. BMI, body mass index; 95% CI, 95% confidence interval; DBP, diastolic blood pressure; HOMA-IR, homeostatic model assessment for insulin resistance; MAP, mean arterial pressure; SBP, systolic blood pressure; |t|, T statistic.

## DISCUSSION

Obesity is characterized by chronic, low-grade inflammation which affects many tissues including skeletal muscle. Skeletal muscle inflammation is a driver of insulin resistance, and exercise is an effective countermeasure to mitigate skeletal muscle inflammation and insulin resistance in the context of obesity (69). We have for the first time demonstrated that the inflammatory receptor RAGE is upregulated in skeletal muscle from individuals with obesity, which may be contributing to the inflammatory state of obesity as indicated in the current work by elevated levels of skeletal muscle P65 gene expression. Contrary to our hypotheses, RAGE alternative splicing in the muscle was not affected by obesity or acute AE. In addition, individuals with obesity did not possess lower circulating sRAGE isoforms, and acute AE was not effective at augmenting circulating esRAGE or cRAGE isoforms.

Our laboratory and others have previously demonstrated a strong negative relationship between obesity and sRAGE isoforms (17, 42, 62, 64, 65). In particular, we have found that esRAGE tends to be more strongly correlated than cRAGE to indices of obesity such as BMI and body fat percentage (42) and is most responsive to weight loss (62). However, in this cohort, the sRAGE isoform concentrations in the LH and OB groups were equivocal, and sRAGE was not correlated with BMI or body fat percentage (Supplemental Table S1). This discrepant finding with the literature is most likely due to the low sample size of our cohort (*n* = 24) and in particular, our OB group (*n* = 7). In addition, individuals in our OB group were mildly obese (class I), whereas previous investigations, including our own, which have demonstrated lower circulating sRAGE isoforms in individuals with obesity have studied individuals that would be characterized as having class II or III obesity based on their BMI (10, 42, 65, 82, 83). In addition, the participants in the current study may lack aspects of the obesogenic milieu that drive these relationships. For example, we do not have data on how long these individuals were classified as obese, which has been demonstrated to track the development of metabolic dysfunction (84). Individuals in the OB group possessed significantly elevated fasting insulin and HOMA-IR but had similar fasting blood glucose compared with the LH (Table 1). This phenotype is characteristic of the early stages of the natural history of diabetes (67). It is conceivable that this stage of the natural history of diabetes precedes a decrease in circulating sRAGE. In other words, obesity alone may not be sufficient to suppress circulating sRAGE, or this effect may occur in more severe cases of obesity and metabolic dysfunction. Indeed, we previously showed that lower sRAGE concentration correlated with severity of obesity and glucose tolerance (42). In further support of this notion, our multiple regression analysis in this current investigation indicates that insulin sensitivity assessed by HOMA-IR was a significant predictor of increased skeletal muscle RAGE abundance (Table 3). In addition, although not significant, there was a similar trend for HOMA-IR to predict variance in RAGE transcripts as well (β = 90.72, *P* = 0.1282).

To our knowledge, this is the first investigation to examine the effect of acute AE intensity on sRAGE production and to explore the effect of exercise on sRAGE isoforms in lean, healthy individuals or individuals with obesity. Based on prior knowledge of sRAGE-producing mechanisms such as cleavage of RAGE by ADAM10 in other cells and tissues, we hypothesized that acute AE would promote an increase in sRAGE isoforms in an intensity-dependent manner. However, while the change in sRAGE isoforms with exercise tended to be negative on average, the effect size was small and increased exercise intensity tended to diminish this effect. However, the small or nonexistent effects of exercise on sRAGE may be due in part to the individuals with obesity having similar (and presumably optimal) levels of sRAGE as the LH group at baseline. Future work should examine if exercise produces a more robust effect in individuals starting with suboptimal sRAGE values as well as explore some of the other mechanisms by which exercise may promote sRAGE production.

Given the involvement of skeletal muscle in exercise and the relatively large proportion of body mass that is muscle, we hypothesized that muscle would be a primary contributor to the circulating sRAGE pool with exercise and that this would be reflected in the muscle by lower RAGE expression. However, exercise had no effect on RAGE protein or gene expression in the muscle from the LH or OB group. This finding was consistent when examining the effect of acute exercise on TLR4, which is known to heterodimerize with RAGE upon activation by certain ligands (81) and has recently been demonstrated to be responsive to acute exercise (85). Like RAGE, TLR4 is also able to be cleaved by ADAM10 to form soluble TLR4 (86). The lack of change in muscle RAGE is in line with our observations of sRAGE being minimally affected by the acute exercise in this cohort. One explanation for this finding is that the turnover of RAGE is much slower than we anticipated, and therefore, our sampling window was unable to detect these changes. A lack of change in muscle RAGE with exercise could also suggest that the changes in sRAGE are likely attributable to the effects of exercise on other cells and tissues. RAGE is highly expressed in many other cells including endothelial cells, adipose, and immune cells all of which are differentially affected by exercise. For example, acute exercise induces inflammation in part via phenotype switching of monocytes to a more inflammatory phenotype (87). Interestingly, monocytes and neutrophils treated with sRAGE promote heterodimerization of sRAGE with RAGE on the membranes of monocytes and instigate inflammatory cytokine production (88). Perhaps, acute exercise promotes sRAGE to be sequestered by circulating monocytes until this is outpaced by sRAGE production. Together, these data might suggest that acute aerobic exercise at 80% of V̇o_2max_ is not sufficient to adequately activate ADAM10 to promote cleavage of RAGE or TLR4 protein or accretion of circulating sRAGE isoforms in this relatively healthy cohort lacking an overt inflammatory phenotype.

Previous work by our laboratory demonstrated circulating esRAGE as the strongest sRAGE isoform correlated with BMI (42) and most responsive to weight loss (62). Therefore, we hypothesized that RAGE alternative splicing would be dysregulated to favor RAGE expression while forsaking esRAGE alternative splicing with obesity. Interestingly, individuals with obesity possessed higher RAGE protein and esRAGE transcripts than LH group. However, esRAGE transcripts were also elevated in OB muscle samples and correlated with BMI (ρ = 0.486, *P* = 0.03) and body fat percentage (ρ = 0.640, *P* = 0.002) (Supplemental Table S1), suggesting that RAGE gene transcription is elevated and that the ratio of splicing full-length RAGE to esRAGE was not affected by obesity. Given the known issues with targeted short PCR and the complex regulation of RAGE transcription, this finding should be validated in future studies using long-read sequencing.

RAGE drives inflammation through NF-κB which promotes further RAGE expression. Transcripts for P65 were also elevated in individuals with obesity and correlated with BMI and body fat percentage (Supplemental Table S1), suggesting that this feed-forward mechanism is able to promote RAGE transcription without altering the alternative splicing regulation. This interpretation is further supported by correlations with RAGE and esRAGE transcripts with NF-κB. Correlations of RAGE protein and transcripts with clinical outcomes such as BMI, body fat percentage, and lean mass demonstrate the negative implications of exacerbated RAGE expression in muscle. In fact, RAGE protein has been previously suggested to not be present in mature muscle cells unless they are severely damaged such as in the context of muscular dystrophy (89). RAGE’s physiological functions in muscle appear to be related to muscle development following which transcripts become downregulated upon differentiation of myoblasts (90). However, we have clearly observed RAGE at both the transcript and protein levels in muscle from young healthy individuals using an antibody that has been knockdown validated via siRNA in our hands (Supplemental Fig. S3). Regardless, the exacerbated expression of RAGE in muscle from individuals with obesity are likely a maladaptation that may play a pathogenic role related to obesity. Indeed, genetically modified animals lacking muscle RAGE gain protection from a number of pathogenic consequences in muscle including ischemia reperfusion injury (16) and muscle dystrophy (91).

### Summary

In summary, these data suggest that RAGE expression is exacerbated in muscles in the early stages of obesity and may contribute to the chronic low-grade inflammatory phenotype that persists in this context. Contrary to our hypothesis, AE had no effect on muscle RAGE protein abundance or RAGE splicing and tended to provoke a small decrease in sRAGE at low and moderate exercise intensities. Future work should continue to explore the underlying mechanisms and consequences of exacerbated RAGE expression in muscle or other tissues and determine if other interventions that alter energy balance such as caloric restriction alone or in combination with acute or chronic exercise are able to rescue this phenotype.

## DATA AVAILABILITY

Data will be made available upon reasonable request.

## SUPPLEMENTAL DATA

10.6084/m9.figshare.23519022.v2 Supplemental Tables S1 and S2 and Figs. S1–S5: https://doi.org/10.6084/m9.figshare.23519022.v2.

## GRANTS

This work was supported by an American Diabetes Association-Junior Faculty Award 1-14-JF-32 (J.M.H.) and NIH R01 DK109948 (J.M.H.).

## DISCLOSURES

No conflicts of interest, financial or otherwise, are declared by the authors.

## AUTHOR CONTRIBUTIONS

E.R.M., A.T.L., and J.M.H. conceived and designed research; E.R.M., J.T.M., B.K.B., A.B.C., K.N.Z.F., and J.M.H. performed experiments; E.R.M., A.T.L., and J.M.H. interpreted results of experiments; E.R.M. prepared figures; E.R.M. drafted manuscript; E.R.M., J.T.M., B.K.B., A.B.C., K.N.Z.F., R.K.P., A.T.L., and J.M.H. edited and revised manuscript; E.R.M. and J.M.H. approved final version of manuscript.
